# Gas exchange, vine performance and modulation of secondary metabolism in *Vitis vinifera* L. cv Barbera following long-term nitrogen deficit

**DOI:** 10.1007/s00425-021-03590-8

**Published:** 2021-02-22

**Authors:** Cecilia Squeri, Begoña Miras-Moreno, Matteo Gatti, Alessandra Garavani, Stefano Poni, Luigi Lucini, Marco Trevisan

**Affiliations:** 1 Department of Sustainable Crop Production, Università Cattolica del Sacro Cuore, Via Emilia Parmense, 84, 29122 Piacenza, Italy; 2 Department for Sustainable Food Process, Università Cattolica del Sacro Cuore, Via Emilia Parmense 84, 29122 Piacenza, Italy

**Keywords:** Metabolomics, Nutrient deprivation, Strigolactones, Photosynthesis, Grape ripening, yield

## Abstract

A reprogramming of secondary metabolism to acclimate to nitrogen deficiency was seen in grapevine eliciting an accumulation of strigolactones and jasmonate. This response links with photosynthetic compensation and enhanced ripening.

In addition to the metabolism directly related to nitrogen assimilation, long-term nitrogen depletion may affect plant secondary metabolism, in turn affecting grapevine performance. In this work, the effect of nitrogen deficit was investigated in *V. vinifera* cv. Barbera potted vines following three years of deprivation, using a combination of morpho-physiological assessments and mass spectrometry-based untargeted metabolomics. Plants grown under nitrogen limitation showed reduced growth and even more curtailed yields, lowered SPAD values, and a quite preserved leaf gas exchange, compared to plants grown under non-limiting nitrogen availability. Ripening was decidedly accelerated, and berry composition improved in terms of higher sugar and phenolic contents under nitrogen-limiting conditions. Metabolomics showed the broad involvement of secondary metabolism in acclimation to nitrogen deficiency, including a distinctive modulation of the phytohormone profile. Several nitrogen-containing metabolites were down accumulated under nitrogen-limiting conditions, including alkaloids, glucosinolates, hypoxanthine, and inosine. On the other hand, phenylpropanoids showed an accumulation trend. Concerning the recruitment of hormones, nitrogen deprivation elicited an accumulation of strigolactones and jasmonate. Noteworthy, both strigolactones and jasmonates have been previously related to increased photosynthetic efficiency under abiotic stress. Furthermore, the severe reduction of lateral shoot development we recorded in N-deprived vines is consistent with the accumulation of strigolactones. Overall, our results suggest that nitrogen deprivation induced a rather broad metabolic reprogramming, mainly including secondary metabolism and hormones profile, reflected in the modulation of photosynthetic performance, canopy growth, and possibly fruit quality.

**Supplementary Information:**

The online version contains supplementary material available at 10.1007/s00425-021-03590-8.

## Introduction

Despite the considerable know-how and progress made over the last decades in vine physiology and cultural practices (Poni et al. 2018; Jamali et al. 2020), a large number of poorly explained or unknown cause-effect relationships between environmental and agronomical inputs and outputs does exist especially in the realm of grape production. The degree of plasticity of a given genotype to adapt to the new environment is still poorly understood (Webb et al. 2012), and the success or failure of the different approaches is still mostly dictated by empirical or, even worse, anecdotal evidence (Rossouw and Bauer 2009).

Vine nutrition is an important factor for optimal vine balance and desired wine properties and, among essential vine nutrients, nitrogen (N) is the most abundant soil-derived macronutrient in grapevine and plays a pivotal role in many biological processes related to vine and berry growth, berry composition, and fermentation kinetics (Bell and Henschke 2005). While it has been ascertained that N seasonal peak demand in the grapevines occurs around the flowering-fruit set (May 2004), growers still encounter difficulties at matching demand with supply at specific phenology stages. Depending on the amount, timing, and N form used within a given fertilization strategy (Lang et al. 2020), N availability can either be too high early in the season when N demand is largely met by the reserves or, vice versa, N becomes available too late in the season (i.e., after fruit set) therefore pushing vegetative growth when the reproductive phase should instead be prioritized.

N-deficiency is especially harmful when occurring around flowering (May 2004). It can severely curtail the current season fruit-set while also having adverse effects on next-year bud induction (Guilpart et al. 2014). N excess can likely be even more detrimental as it can lead to excessive vigor and consequent ripening delay, poor wood maturation and next season bud induction and differentiation (Mendez-Costabel et al. 2014; Mundy, 2008). Furthermore, it compacts clusters with large berries and less favourable skin-to-berry ratio (Gatti et al. 2017) and downregulates genes involved in anthocyanin biosynthesis (Soubeyrand et al. 2014). N is also involved in the leaf longevity process, as it has been demonstrated that photosynthetic decline in a mature grapevine leaf is linked to increased N export towards growing sinks (Poni et al. 1994; Pyung et al. 2007).

It is well known that nitrate requires two reduction steps (nitrate reductase in the cytosol using NADH, and nitrite reductase in the chloroplast) before being incorporated by the glutamine synthetase—glutamate synthase (GS-GOGAT) pathway (Beevers, 1981). Unlike this well-consolidated knowledge, comparatively less information is available on long-term effects related to N availability. When it comes to the relationship between nitrogen supply/demand and vine behaviour, grape composition and wine properties, metabolomics and biochemical information are almost totally lacking. The only exception is a recent paper by Lang et al. (2019), who analysed the leaves and wines’ metabolic patterns obtained from the cv. Regent coming from N fertilization with different N forms, as compared to unfertilized control. Interestingly they found that the total number of metabolites found in leaf and wine samples was significantly changed by the N form, even though the treatments were receiving the same amount of N (60 kg/ha) and that the non-fertilized plots likely were not showing any N-deficiency.

The objectives of this study were to: (i) investigate the metabolic signatures of *V. vinifera* cv. Barbera leaves sampled from potted vines subjected to N fertilization or deprived of any N supply and assess how consistent is the metabolic reprogramming, and (ii) establish any link between the observed metabolic patterns and leaf physiology parameters, vine performance, and grape ripening. The latter objective is especially novel as it combines multidisciplinary surveys aiming at clarifying if and why a severe N deficiency might differentially affect yield components and final grape composition. Hypothesis is made that no or limited N supply might severely impair vegetative growth and yield, while at the same time resulting in accelerated ripening due to physiological and metabolome-related compensation mechanisms. Results are expected to be relevant within a global warming scenario, where any agronomic tool available to regulate the dynamics of berry ripening is very much needed.

## Materials and methods

### Plant material and experimental layout

The trial was carried out in 2018 at the Department of Sustainable Crop Production (DIPROVES) of the Università Cattolica del Sacro Cuore (45°02′ N, 9°43′ E, 54 m asl) using a batch of 20 five-year-old cv. Barbera vines grafted onto 110 Richter rootstock (*V. Berlandieri* × *V. Rupestris)* grown outdoor in 14 L pots filled with loamy soil with available water of 110 mm/m. Soil chemical-physical properties are reported in Table S1, and main characteristics were as follows: 12.1% and 24.4% and 1.46 g/cm^3^ corresponding to the wilting point, the field capacity and the bulk density, respectively, according to Saxton and Rawl (2006). Weather trends of the growing season are shown in Fig. S1.

Plants were head-trained and two 2-node spurs per vine were kept at winter pruning. Two weeks after budburst, estimated as reaching the swollen bud stage, vines were thinned to four main shoots. Eight vines were assigned to an N-fertilized (N +) treatment from the initial group, whereas an equal number of vines was kept as an unfertilized N control (N0). Differential fertilization was performed in N + plants using ammonium nitrate (34% N) at a dose of 0.5 g per application. Phosphorus and potassium were provided to both N + and N0 vines using a 0:52:34 (N:P:K) fertilizer at the rate of 0.5 g per application. Fertilization was carried out manually by dissolving fertilizers in 1 L of water per vine and watering the pot's top with the solution. Applications were performed three times per week between inflorescence swelling and the beginning of ripening in 34 fertilization events. To increase the probability of incurring severe N deficiency, the same fertilization layout was applied in 2016 and 2017, resulting in a total of 30 and 39 fertilization events, respectively. Pots were painted white to minimize possible overheating of the root system and irrigated twice a day to prevent any water stress while assuring the best condition for N uptake. Underpots were used to avoid water percolation.

### Vine measurements

On each vine (eight per treatment), two shoots (S1 and S2) were selected in spring, and three leaves were tagged on S1 to represent basal (4–5th node, cluster zone), median (8–9th node), and apical (11–12th node) shoot zones. On each leaf, the Greenness Index was determined by using a SPAD-502 portable meter (Konica Minolta, Osaka, Japan). For each leaf, five determinations were taken and averaged considering main and lateral lobes. SPAD readings were taken on five dates along the season, corresponding to DOY (Day of Year) 128, 152, 166, 177, and 201, covering a leaf age span of 73 days.

At four of the same five dates and on the same leaves, the SPAD index was measured, leaf assimilation (A, µmol m^−2^ s^−1^) and stomatal conductance (g_s_, mmol m^−2^ s^−1^) were measured with the portable gas-exchange analyzer LCi-SD (ADC BioScientific Ltd., Hoddesdon, UK). All readings were taken in the morning hours (10:00–13:00) under a clear sky and saturating light conditions. Previous studies have demonstrated that N deficiency exhibits different patterns depending on leaf age (Poni et al. 1994), hence the evaluation of physiological data against leaf position is also recommended. Measurements were taken at ambient relative humidity, and the flow fed to the broad-leaf chamber (4.5 cm^2^ window size) was 300 mL min^−1^.

At veraison (July 27), leaves from nodes 4, 7, and 10 along the stem were sampled from all S1 shoots. Thus, for each treatment × node position combination, eight leaves were harvested and pooled in a composed sample. Each leaf was promptly weighted, and blade surface was determined through a leaf-area meter (LI-COR 3000 Bioscience, Lincoln, NE). After that, petioles were removed, and leaf blades were washed twice in distilled water, dried in a forced-air oven at 75 °C, finely ground, and sent to an external laboratory for mineral ion analysis. The same sampling procedure was performed on S2. Sampled leaves were then immediately frozen and stored at − 80 °C in 50 mL Falcon tubes until subsequent metabolomic analysis.

At harvest, occurring on DOY 234 (August 22) for both treatments, all clusters were individually picked, immediately weighed, and taken to the laboratory. Clusters from S1 and S2 were then destemmed, berries were counted and classified as a function of their size (i.e., normal and shot berries). Accordingly, cluster compactness was evaluated as cluster mass-to-rachis length. A sub-sample of 10 intact berries per cluster was taken and stored at − 18 °C to determine the berry mass and individual berry organs (skin, flesh, and seeds). Skin, flesh, and seed mass were then determined by using a razor blade and a small metal spatula. Seeds and flesh were carefully removed from each berry without rupturing any pigmented hypodermal cells, then the seeds carefully separated from the flesh with tweezers and the number of seeds per berry was counted. Both skins and seeds were rinsed in deionized water, blotted dry, and weighed. A second sub-sample of 50 healthy berries was stored at − 18 °C and then used for the determination of anthocyanins and total phenolic concentration, according to Iland et al. (2011), as well as for flavonols and single anthocyanidins by HPLC.

Remaining berries were crushed, and the resulting juice immediately processed to determine TSS using a temperature compensated refractometer whilst titratable acidity (TA) was measured by titration with 0.1 N NaOH to a pH 8.2 endpoint and expressed as g/L of tartrate equivalents. Must pH was measured simultaneously using a pH-meter CRISON GLP 22 (Crison, Barcelona, Spain).

The concentration of tartaric and malic acids was determined by HPLC. The quantification of organic acids was performed by injecting 1:4 diluted must into HPLC after filtering through a 0.22 μm polypropylene filter. The identification was performed by external calibration with standards, and concentration was calculated measuring the peak area and expressed in g/L. For this analysis, an Allure Organic Acid column, 300 × 4.6 mm, 5 µm (Restek, Bellefonte, PA, USA) was used. The separation was performed in isocratic conditions using water, pH was adjusted at 2.5 by adding orthophosphoric acid and injection volume was 15 µL. The elution was monitored at 200–700 nm and detected by UV–Vis absorption with DAD at 210 nm.

The berry samples were manually and carefully peeled, and the resulting skins and seeds were immediately freeze-dried. Phenolic compounds were extracted from skins after Downey and Rochfort (2008): 0.100 g of freeze-dried skin were extracted in 1.0 mL of 50% (v/v) methanol in water for 15 min with sonication (Downey and Rochfort, 2008). The phenolic compounds of seeds were extracted after Poni et al. (2017) with a minor modification: 0.200 g of freeze-dried seeds were extracted in 25 + 5 mL of methanol/ethanol (8:2, v/v) by sonication, concentrated in a rotavapor, then resuspended and filtered through a 0.22 µm polypropylene syringe into glass vials for HPLC–DAD analysis.

An Agilent 1260 Infinity Quaternary LC (Agilent Technology, Santa Clara, CA, USA) equipped with a reverse-phase C-18 Synergi Hydro RP 80 A, 250 × 4.6 mm, 4 µm (Phenomenex, Torrance, CA, USA) column was used. A binary gradient elution, with 5% (v/v) formic acid (solvent A) and acetonitrile (solvent B) flowing at 0.5 mL/min, and UV–Vis DAD detection (200–700 nm) were also adopted. Phenolic compounds were identified using authentic standards; however, petunidin 3,5-*O*-diglucoside and acylated or coumarated anthocyanins were identified by comparison to data available in the literature.

At the end of the season, immediately after leaf fall, node counting was performed on either main and lateral canes. Thereafter, the one-year wood's pruning weight was recorded by separating the two wood types. Main total leaf area (LA) was then calculated from node counts and mean leaf surface recorded prior to nutritional assessment. For laterals, leaf area was estimated from already available allometric relationships between leaf area and fresh weight derived from a five-year study conducted on the same cultivar (Bernizzoni et al. 2009).

### Metabolomic analysis

Metabolomic analysis was carried out as previously reported (Salehi et al. 2018). Frozen leaves originally taken from S2 shoots (8 leaves per treatment × node position combination) were homogenized in liquid nitrogen using pestle and mortar, and then an aliquot (1.0 g) was extracted in 10 mL of 0.1% HCOOH in 80% methanol using an Ultra-Turrax (Ika T-25, Staufen, Germany). The extracts were centrifuged and then filtered through a 0.22 μm cellulose membrane into glass vials for analysis. The metabolomic analysis used liquid chromatography quadrupole-time-of-flight mass spectrometry (UHPLC/QTOF). The QTOF operated in positive polarity SCAN mode (100–1200 m/z range). The chromatographic separation was achieved using a binary mixture of water and methanol as a mobile phase on a Zorbax Eclipse-plus column (75 × 2.1 mm i.d., 1.8 μm—Agilent Technologies, CA, USA). A binary gradient elution (5% to 90% methanol within 35 min) was also adopted, with a flow rate of 220 μL min^−1^. Post-acquisition processing (deconvolution, mass and retention time alignment) and compounds annotation were carried out using the software Profinder B.07 (from Agilent Technologies) as previously reported (Rocchetti et al. 2019). Features were filtered by frequency: the compounds not present in 66% of replications within at least one treatment were discarded. Compound annotation was finally achieved by combining monoisotopic accurate mass, isotope spacing, and isotope ratio versus the database exported from PlantCyc 9.6 (Plant Metabolic Network, http://www.plantcyc.org) according to (Rouphael et al. 2020). Therefore, annotations corresponded to Level 2 of COSMOS Metabolomics Standards Initiative (Salek et al. 2015).

### Statistical analysis

Plant growth, yield components, nutritional status, and fruit composition data were subjected to a one-way analysis of variance (ANOVA) and N + versus N0 comparisons performed by *t* test at *p* < 0.05. Vine replicates were 8 per treatments and sub-replicates were the two tagged shoots per vine. Nutritional status was assessed on three replicates per treatment corresponding to basal, median and apical leaves.

Repeated measures of SPAD units and gas exchange parameters taken along the season were analyzed with the Repeated Measure Analysis of Variance (ANOVA) routine embedded in the XLSTAT software package (Addinsoft, Paris, France). The least squared (LS) mean method at *p* =  < 0.05 was used for multiple comparisons within dates. Equality of variances of the differences between all possible pairs of within-subject conditions (i.e*.*, levels of vigor or fertilization strategy) was assessed through Mauchly’s sphericity test.

Regarding metabolomics, for each treatment, three replicates corresponding to basal, median and apical leaves were analyzed (for a total of 6 samples). Compounds abundance was log2 transformed and normalized at the 75th percentile. Thereafter, unsupervised hierarchical cluster analysis (HCA, Squared Euclidean distance) was built, based on fold-change values, using Mass Profiler Professional B.07 (Agilent Technologies). OPLS-DA supervised analysis was next carried out in SIMCA 16 (Umetrics, Malmo, Sweden) at default parameters. Outliers were excluded using Hotelling’s T2 (95% and 99% confidence limit for the suspect and strong outliers). The supervised model was validated (CV-ANOVA, *p* < 0.01), permutation testing (*n* = 100) used to exclude overfitting, and goodness-of-fit R2Y and goodness-of-prediction Q2Y calculated. After that, Variables Importance in Prediction (VIP analysis) allowed identifying the most discriminant compounds, and then Volcano Plot analysis (ANOVA with *p* < 0.01 and Bonferroni multiple testing correction, followed by fold-change analysis with a threshold of 2) was carried out to identify the significant metabolites. These latter compounds were finally interpreted in the Omic Viewer Pathway Tool of PlantCyc to identify pathways and biochemical processes affected by low nitrogen availability (Caspi et al. 2013).

## Results

### Leaf greenness and gas exchange

**Fig. 1:**
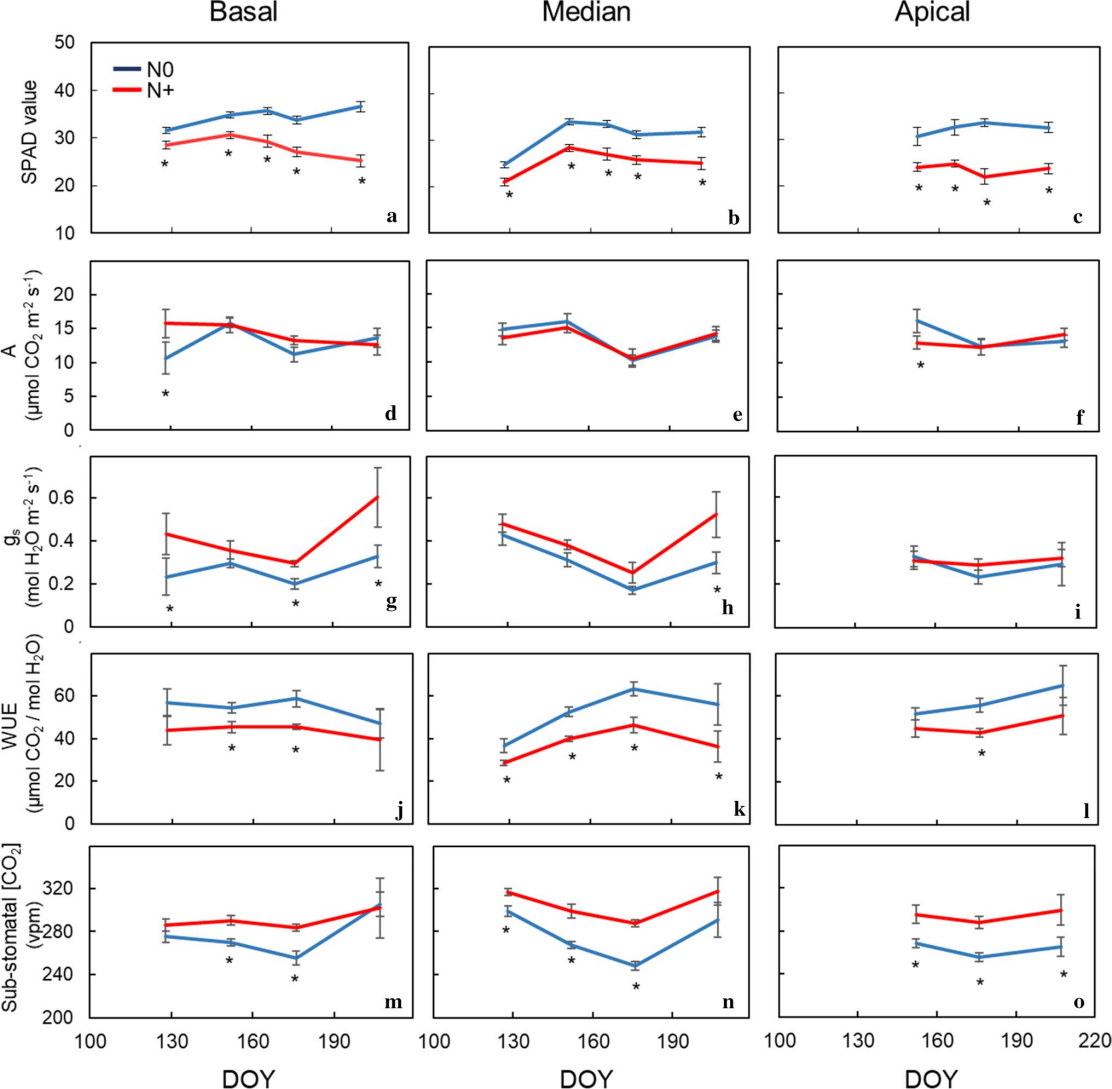
Seasonal variation of the Greenness Index (SPAD) (**a**–**c**), leaf assimilation (**d**–**f**), leaf stomatal conductance (gs) (g_s_), intrinsic WUE (A/g_s_) (**j**–**l**) and sub-stomatal CO_2_ concentration (c_i_) (M–O measured on basal, median and apical leaves of Barbera vines subjected to different nitrogen fertilization (N + versus N0) in 2018. First fertilization: 7 May 2018 (DOY 127). Within each sampling date, the asterisk indicates significant differences between N supply at *p* < 0.05 according to *t* test. Vertical bars indicate standard error (SE). DOY, Day of Year

Greenness index (taken with the SPAD 502 Minolta meter at five dates from mid-May until mid-July on basal, median, and apical leaves of the two treatments) showed higher values in N + than N0 regardless of the date and the position considered (Fig. 1a–c). Conversely, at most measuring dates and positions on the shoot stem, leaf assimilation rates (A) did not differ between the two levels of N supply (Fig. 1d–f). When SPAD and A rates data taken on the same leaves were pooled over different sampling dates and shoot positions, the resulting correlation was not significant (data not shown). Different N supply impacted leaf g_s_ in basal and median shoot portions, with N deprivation inducing higher g_s_ in three out of four measuring dates (Fig. 1g, h). Vice versa, leaf g_s_ measured on apical leaves differed between treatments only at the intermediate sampling date (Fig. 1i). As a result of relative variation of A and g_s_, intrinsic WUE (A/g_s_) of N + leaves significantly diminished at any date in basal and median shoot portions, whereas the trend was milder in apical leaves (Fig. 1j–l). Sub-stomatal [CO_2_] resulted to be always higher in N0 regardless of sampling dates and leaf position on the stem (Fig. 1m–o).

### Vine performance

No N supply was effective in determining a significantly lower N blade concentration in N0 leaves at veraison, which also matched with higher P, Mn and Zn concentrations (Table 1). In N0, vegetative growth as total one-year-old pruning weight was reduced by 38%, albeit fractional limitations were much higher for lateral canes (− 74%) than for main canes (− 27%). A similar outcome applied to leaf area per vine data, where the lateral contribution was reduced by 65% in N0 vs N + , whereas total leaf area per vine diminished by 33% in N0 as compared to the N fertilised control.

**Table 1:** Leaf nutrition; vegetative growth, yield and berry growth components and grape composition in vines that, starting from 2017 and until the trial year (2018), were subjected to N supply (N +) or received no N supply (N0)

		N +	N0	Sig.
N	(%)	2.18	1.66	*
P	(%)	0.35	0.64	**
K	(%)	0.71	0.70	ns
Ca	(%)	2.97	3.27	ns
Mg	(%)	0.72	0.66	ns
S	(%)	0.18	0.18	ns
Na	(ppm)	110	115	ns
Fe	(ppm)	99	96	ns
Mn	(ppm)	48	65	*
B	(ppm)	20	20	ns
Zn	(ppm)	17	20	**
Main pruning weight	(g/vine)	233.6	170.0	*
Lateral pruning weight	(g/vine)	72.9	19.3	**
total pruning weight	(g/vine)	306	189	**
Main leaf area	(m^2^/vine)	1.28	1.05	*
Lateral leaf area	(m^2^/vine)	0.59	0.16	*
Total leaf area	(m^2^/vine)	1.87	1.21	**
Yield	(g/vine)	258	97	**
Cluster weight	(g)	96.1	34.5	*
Berry weight	(g)	1.60	1.31	ns
Berries/cluster	(n)	60.1	26.3	**
Cluster compactness	(g/cm)	10.61	4.85	*
Shot berries	(%)	9.62	22.26	*
Skin weight	(g/berry)	0.15	0.13	**
Flesh weight	(g/berry)	1.53	1.12	**
Total seed weight	(g/berry)	0.08	0.05	**
Mean seed weight	(mg)	35.65	31.69	**
Seed number	(n/berry)	2.30	1.53	**
Skin-to-berry ratio	(%)	9.11	10.26	ns
Seed-to-berry ratio	(%)	4.69	3.83	**
Flesh-to-berry ratio	(%)	86.21	86.17	ns
Skin-to-flesh ratio	(%)	10.68	12.10	ns
Total soluble solids	(Brix)	24.17	26.30	*
ph		3.46	3.39	ns
Titratable acidity	(g/L)	13.91	9.25	**
Tartrate	(g/L)	5.60	7.19	*
Malate	(g/L)	6.44	3.18	**
Total anthocyanins	(mg/g)	0.346	1.049	**
Total phenols	(mg/g)	2.250	3.258	**

Nutrition assessment was performed on leaf blades sampled at veraison (27 July) from different node position along the main stem (basal, median and apical)

Within a row, mean separation was performed by *t* test. *p < 0.05, **p < 0.01, ns not significant

Yield per vine was also severely curtailed by N starvation as in N0 reduction was -63% *vs*. N + with cluster weight and, namely, the component of berries/cluster, registering the most severe limitation. The consequence of reduced berry size and berries/cluster in N0 was a much looser cluster. However, such a feature was likely contributed also by a higher fraction of shot berries reaching 22.3% over total berry number in N0 vs. 9.6% detected in N + . Absolute growth of single berry organs (skins, flesh, seed meant either as the total weight and number/berry) were all limited by N starvation; however, when data were given on a relative basis, most of such differences vanished, and only a reduced seed-to-berry ratio (fresh weight basis) remained (Table 1). Computing source-to-sink balance as leaf area-to-yield ratios (cm^2^/g) showed that source availability for ripening increased in the N0 treatment reaching 130 cm^2^/g of fresh fruit mass against 72 cm^2^/g calculated for the N + vines.

Grape composition at harvest was significantly impacted by N supply (Table 1). Ripening was decidedly accelerated by N starvation as TTS concentration (+ 2.1 Brix than N +), lower TA and malate and very much improved phenolic maturity with total anthocyanins and phenols concentration increasing by 303% and 44.8%, respectively. The concentration of each of the five glucoside anthocyanins was also greatly enhanced and a similar effect was seen for flavonols with quercetin 3-*O* glucoside peaking at 0.880 mg/g (Table S2). Interestingly, N0 vines maintained higher tartaric acid concentration than N + at harvest.

### Metabolomic profiling

**Fig. 2:**
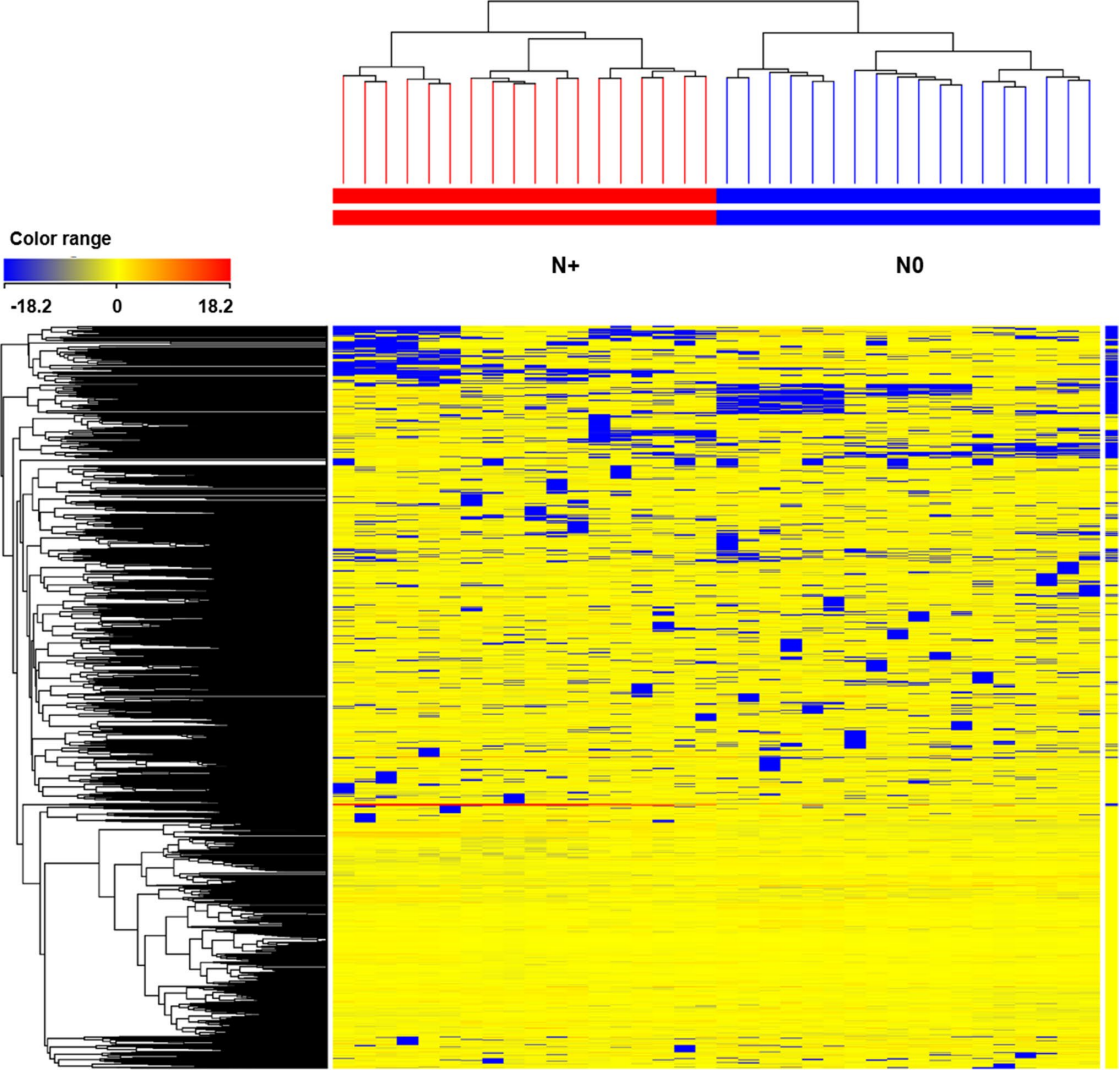
Unsupervised hierarchical clustering of the metabolic profile of leaves of Barbera vines subjected to different nitrogen fertilization. Samples (each different biological replicate per condition) are identified by colored segments of the top-bars. A fold-change based heatmap was built and samples were clustered with Ward’s algorithm, based on Squared Euclidean distances

**Fig. 3:**
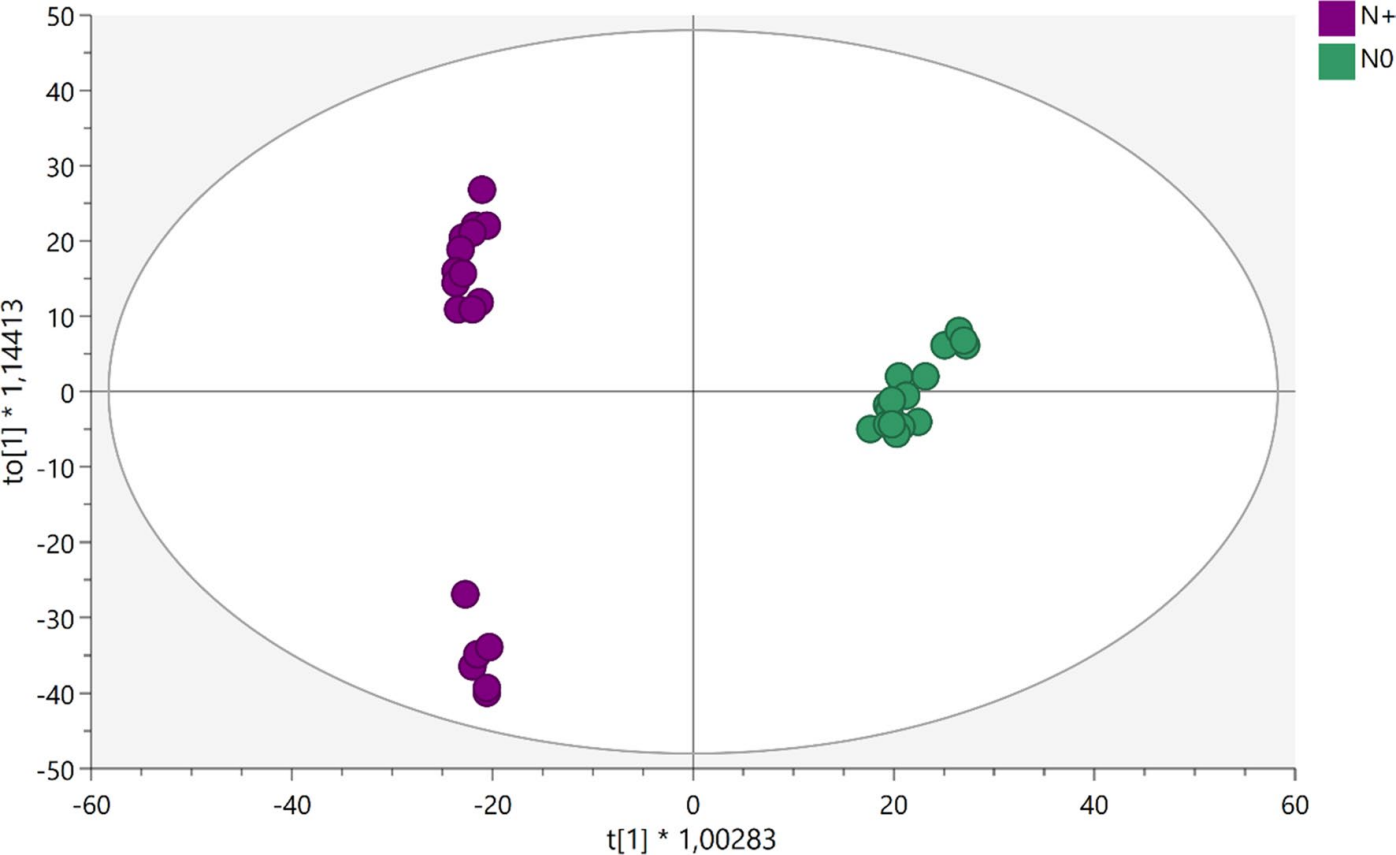
Score plot of OPLS-DA supervised modelling carried out on untargeted metabolomics profiles of leaves of Barbera vines subjected to different nitrogen fertilization

Untargeted metabolomics analysis was then carried out to gain insights into the physiological processes affected by N deficiency. Overall, more than 3600 metabolites were putatively annotated and used for the chemometric interpretation (Supplementary Table S2). The multivariate statistical analysis revealed that N supply broadly affected the metabolic profiling of vine leaves. The unsupervised hierarchical cluster analysis (HCA), based on the fold-change heatmap, showed a clear separation of the samples with two distinct clusters based on the N supply (Fig. 2). The supervised OPLS-DA allowed us to separate the samples in the score plot hyperspace according to N supply (Fig. 3), in agreement with HCA (Fig. 2). The OPLS model exhibited adequate goodness-of-fit (R2Y > 0.989) and prediction ability (Q2Y > 0.94), with a cross-validation CV-ANOVA *p* value < 0.05. To identify the metabolites having a pivotal role in such discrimination observed in the OPLS-DA, the variable importance in projection (VIP) analysis was then carried out. Metabolites with a VIP score > 1.5 were considered as discriminant and are provided as Supplementary Table S3. Several small molecules used as substrates or intermediates in a broad series of biosynthetic reactions were found among these markers. Secondary metabolism was also involved in acclimation to N deficiency. In particular, diterpenoids, triterpenoids, and sesquiterpenes were highlighted. Among these compounds, the terpenic hormones gibberellins and brassinosteroids were found as a discriminant in separation. Similarly, a large amount of phenylpropanoids, mainly anthocyanins, flavonoids, and lignans, were confirmed as VIP markers.

**Fig. 4:**
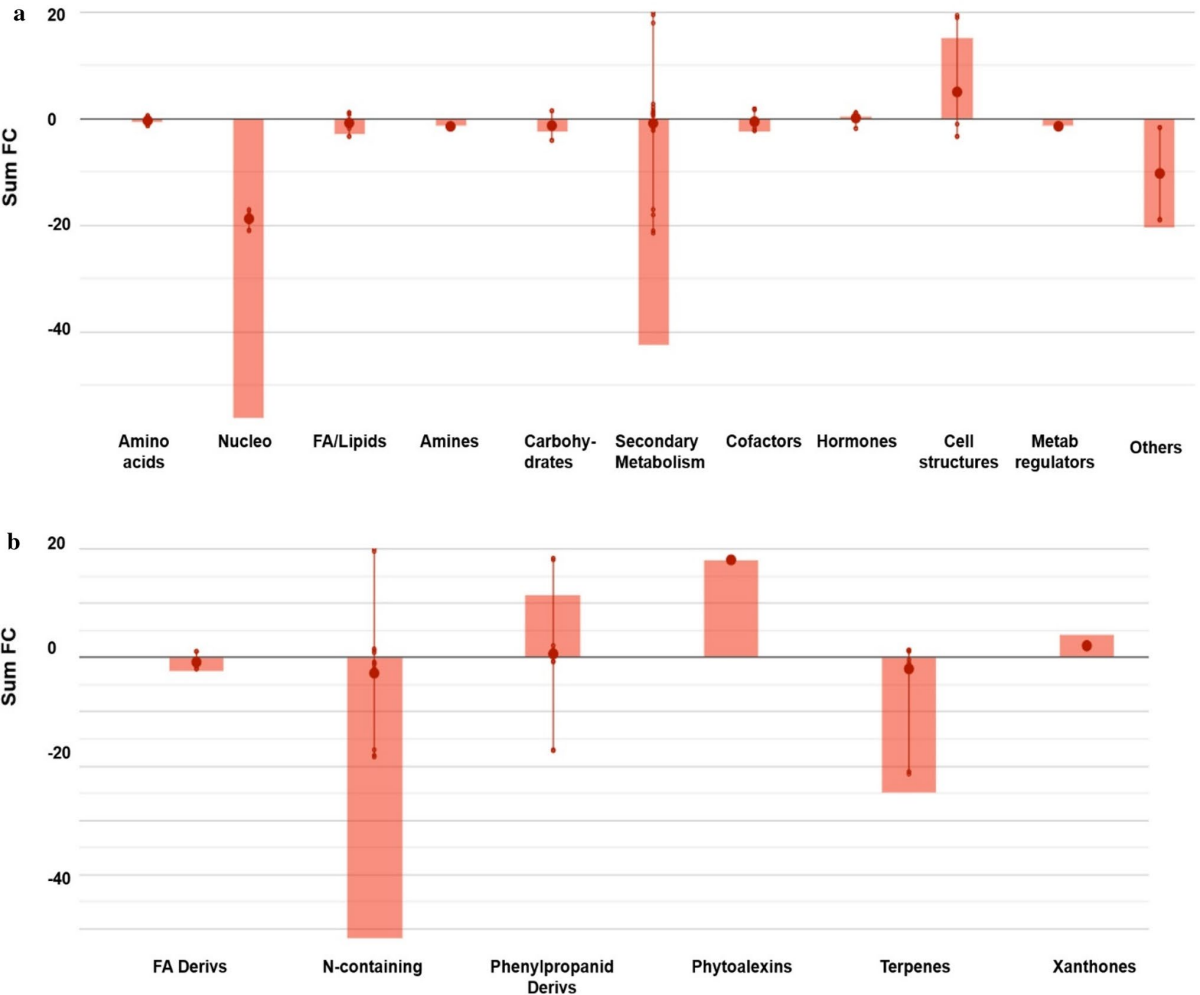
Biological processes (**a**) and secondary metabolism (**b**) affected by N deficiency in leaves of Barbera vines. Differential metabolites and their fold-change (FC) values were elaborated using the Omic Viewer Dashboard of the PlantCyc pathway Tool software (www.pmn.plantcyc.com). In each class, the large dot represents the average (mean) logFC of the metabolites. Small dots represent the individual logFC for each metabolite

A further Volcano Plot analysis was done to strengthen the metabolomic response in grapevine leaves following N deficiency. This resulted in 121 differential compounds (*p* < 0.01, fold-change > 2—Supplementary Table S4), which were used for biochemical interpretation in the omic viewer tool of PlantCyc. The pathway analysis confirmed the role of secondary metabolites in acclimation to nitrogen deficiency (Fig. 4). A rather good agreement was observed between Volcano Plot differential metabolites and OPLS-DA VIP markers of discrimination. Overall, 52 compounds were related to secondary metabolism (Fig. 4b), a generalized down-accumulation trend. Among the metabolites affected by long-term N deprivation, as expected, several N-containing compounds could be identified. Indeed, nucleotides (mostly purine nucleotides and their metabolic products such as hypoxanthine and inosine), *N*-acetyl glucosamine, dopamine quinone, and several glucosinolates (mainly those arising from methionine) were all down accumulated following N deprivation. Furthermore, several compounds related to oxidative imbalance (alpha-tocotrienol, 8-oxo-GMP, octadecadienoate, and hydroxy derivatives of fatty acids) were decreased following N deprivation. On the contrary, an increase of phenylpropanoids, ascribable to the accumulation of conjugates anthocyanins (peonidin derivatives) and lignans, and of 22-oxo-docosanoyl-CoA (precursor of suberin) as well as other acyl-CoA could be observed in response to N-limiting conditions. Moreover, the indole-phytoalexin camalexin, usually elicited by biotic and abiotic stresses, was strongly accumulated. Intriguingly, the concurrent down accumulation of adenosine-5-phosphosulfate (a key compound in sulfate assimilation) and the decrease mentioned above of methionine-derived glucosinolates suggests a cross-involvement of sulfate metabolism.

Concerning phytohormones, a significant modulation was observed due to plant acclimation to limiting N. In particular, the brassinosteroid 6-deoxocastasterone was down accumulated following N deprivation, whereas strigol, the jasmonate precursor 13S-HPODE and auxin conjugates (IAA-Asp, IAA-Gln, and IAA-Glu) showed an opposite trend.

## Discussion

Growing Barbera vines in pots for three consecutive years in a medium deprived of N imposed a significant N deficiency during the trial year (2018). Leaf N concentration assessed pre-veraison in N0 was decreased by 28%, to a leaf concentration of 1.66%. According to literature (Keller and Hrazdina, 1998; Paul Schreiner et al. 2013; Squeri et al. 2019) this value is below the warning threshold of about 1.80% that should be warranted at that phenological stage.

Agronomically speaking, results require a proper interpretation; while the vegetative growth constriction due to inadequate N supply is expected, in our study yield was more than proportionally affected and, as an obvious consequence, the leaf area-to-yield ratio paradoxically increased in the N0 treatment vs. N + (124.7 cm^2^/g vs 72.4 cm^2^/g, respectively). This is a quite perfect match with the ripening dynamic that, even due to this high source availability per unit of the crop, was greatly accelerated in N0 in terms of both sugar concentration and berry pigmentation.

Consistently with the N deficiency status, several nitrogen-containing metabolites were down accumulated in N0 plants, including alkaloids, glucosinolates, as well as hypoxanthine and inosine. Notably, these latter are purine catabolites known to be involved in plant nitrogen metabolism (Brychkova et al. 2008). Indeed, they have been proposed as intermediates to be degraded to urea via uric acid and allantoin, in turn producing ammonia to be re-assimilated via the glutamine oxoglutarate aminotransferase (GOGAT) pathway (Zrenner et al. 2006). Similarly, glucosinolate metabolism has been reported to be largely affected by N deficiency (Zhu et al. 2018). Together with the expected changes in N-containing metabolites, a broad metabolic reprogramming could be observed under N0 in our experiments, to include several other metabolite classes. Cao and co-workers have reported similar results in *I. indigotica*, where both primary metabolites and specialized secondary metabolites were modulated by N starvation (Cao et al. 2020). The effect on metabolite patterns has been recently confirmed in grapevine leaves and has also been linked to wine sensory properties (Lang et al. 2019).

Although having a dramatic effect on both vine performance and grape composition, the metabolic shift we observed under N deficiency had a milder impact on leaf photosynthetic rates. On a methodological basis, it is confirmed that SPAD readings and A rates might show relatively poor correlation, especially when SPAD readings are above the 25 units (Squeri et al. 2019; Steele et al. 2008). The most significant leaf A response observed in our study was that, across leaf position and over a total leaf age span from about 30 to 125 days, N0 maintained A rates quite similar to the N + plants. This confirms grapevine's ability to carry significant photosynthetic compensation under a given biotic or abiotic stress factor (Poni and Giachino, 2000). The degree of stomatal *vs.* non-stomatal limitation is hard to be ascertained (Fila et al. 2006) although, as suggested in previous similar work on grapes by Keller and Hradzina (1988) and Schreiner et al. (2013), non-stomatal limitation seems to be prevalent. In fact, g_s_ was often higher in N0 than N + , in turn leading to lower intrinsic WUE. This agrees with the findings reported by Brueck (2008), reviewing the literature on 15 other species, either field crops and forest trees. In a trial where seedlings of the evergreen perennial *Eucalyptus globulus* L. were grown with five different nutrient treatments embracing a large gradient of N supply, Warren (2004) reported that internal conductance (g_i_) varied between 0.12 and 0.19 mol m^−2^ s^−1^ and the relative limitation of photosynthesis due to internal conductance was more significant than the stomatal limitation. Therefore, our study would confirm that vines kept stomates proportionally more open to maximize A under low N.

However, the degree and extent of A compensation call for other mechanisms which, to our knowledge, have not been previously addressed in grapes. Work carried out by Mu et al. (2016) on maize cultivars having different N tolerance has shown that no effects on A despite specific leaf N content was reduced by 38% (in our study reduction was 28%) and  correlated with higher electron transport rate to maintain stronger PSII activity, which further promoted the ability to harvest and transfer light (Mu et al. 2016). The recruitment of hormones, with an accumulation of strigolactones (SLs) and jasmonate (JA) and, conversely, a down accumulation of brassinosteroids and increased catabolism of auxin, might have contributed to some extent to the photosynthetic performance we observed. In fact, SLs are carotenoid-derived terpene lactone synthesized at the plant root that promote lateral roots and root hair under limited nutrient conditions in an effort to increase the uptake of limited nutrients (Ling et al. 2020). Moreover, SLs are also transported to aboveground organs, where they suppress lateral buds or branches and are able to modify plant response to stresses (Cardinale et al. 2018; Kapulnik and Koltai, 2014; Kohlen et al. 2011). An increased photosynthetic rate was also reported by Min et al. (2019) as a consequence of the foliar application of the synthetic strigolactone rac-GR24 on grapevine seedlings subjected to polyethylene-glycol induced drought stress. Moreover, Zhu et al. (2018) have reported that rac-GR24 ameliorates the adverse effects of drought due to its regulation of stomatal closure and photosynthesis. Accordingly, SLs might have significantly contributed to the improvement mentioned above of stomatal conductance and photosynthetic compensation we observed in N0 leaves. SLs scenario is completed by the severe reduction of lateral development we recorded in our study in N deprived vines. These findings are in agreement with the work by Cochetel et al. (2018) showing that, under N limiting conditions, exudation of SL-like compounds was higher in a rootstock exerting higher control on the vigor of the grafted scion and, namely, in the attitude to branch with the emission of lateral shoots (Cochetel et al. 2018). A concurrent elicitation of methyl jasmonate (MeJA) was observed in N0 plants, compared to well-fertilized control. MeJa is a well-known stress-related hormone, causing a cascade signaling that involves, among others, the activation of programmed cell death (PCD) and defense mechanisms in plants (Creelman and Mullet, 1997). The exogenous application of MeJA to seeds impacted the assembly, stability, and repair of PS II in *B. oleracea* and resulted in an enhanced photosynthetic efficiency (Sirhindi et al. 2020). Such an effect of MeJA showed a dose-dependent relationship and involved PSII efficiency (ɸ PSII) together with photochemical quenching. Furthermore, under light-stress, jasmonate-induced loss of chlorophyll would decrease the amount of energy absorbed by the photosynthetic apparatus, thereby mitigating photochemical damage (Creelman and Mullet, 1997). The concurrent decrease in brassinosteroids and conjugated IAA forms (representing intermediates towards auxin catabolism) is coherent with the reduced SPAD index observed in N0 plants. Indeed, both brassinosteroids and auxins are reported to promote the accumulation of photosynthetic pigments (Hayat et al. 2009; Siddiqui et al. 2018).

Another main finding of our work was that N deficiency strongly impacted fruit ripening by especially promoting anthocyanins and flavonols accumulation in grape berries. At the transcriptomic level, the former effect is not new as N starvation induced upregulation of the phenylpropanoid pathway in Cabernet Sauvignon vines, with delphinidin and petunidin being the most affected compounds (Argamasilla et al. 2014; Soubeyrand et al. 2014). On the other hand, the inverse correlation between N fertilization and phenolics (mainly anthocyanins and flavonols) content is clear, as previously reviewed (Heimler et al. 2017). The accumulation of total anthocyanins recorded in N0 berries does not merely reflect their smaller size (that implies a more favorable skin-to-flesh ratio) since the relative skin growth did not differ between the treatments (Table 1). Such an increase in anthocyanins can be rather directly ascribed to N deprivation, as previously reported in grapevine and other species.

In our study, total flavonols concentration peaked in the N0 treatment, and this throws a challenge about factors involved in such a response. Leaf metabolic profile reported by Lang et al. (2019) related to an unfertilized control and a 60 kg/ha N supply achieved with different N forms led to detect eight significantly changed phenolic components in the leaves, six of which could be assigned to the group of flavonols. Notably, only one phenolic compound increased in abundance compared to the unfertilised control, whereas six flavonols were significantly decreased by any N treatment. However, literature (Brandt et al. 2019; Poni et al. 2018; Price et al. 1995) has clearly shown that berry flavonols, and especially quercetin, behave such as bio-sensors, as being directly involved in sun-screen protection mechanisms under high light conditions. Price et al. (1995) have shown in Pinot Noir that quercetin concentration of an exposed berry can be up to tenfold higher than that recorded on a shaded berry and that this effect also works at local basis meaning that such differences can be found within the berry population forming a single cluster depending on relative light exposure. Under the semi-controlled conditions of our study, differential growth triggered by different N supply has caused significant changes in canopy density hence cluster exposure to light as reflected by different pruning weight and lateral shoot growth (Table 1). Thus, largely improved flavonols concentration determined in N0 grapes might also reflect variation in light microclimate at the cluster level induced by different N supply.

## Conclusions

Metabolic signature assessed in mature leaves sampled from Barbera vines subjected to severe N deficiency has disclosed a broad metabolic reprogramming vs. the normally fertilized treatment encompassing elicitation of phenolics (to include anthocyanins, flavanols, xanthones, lignans, and stilbenes), phytohormones (e.g., strigolactone and jasmonate), the phytoalexin camalexin as well as terpenoids. From one side, some of these changes would directly impinge on final grape composition—enhanced total anthocyanins at harvest in N0 is the ideal example from our study—while also triggering either compensation and defence adjustments. By combining a multidisciplinary assessment of N starvation including metabolomic fingerprint, vine performance and gas exchange we were able to validate the hypothesis that prolonged N deficiency triggers a rather complex compensation scenario allowing maintenance of good leaf assimilation rates while also increasing the source availability for ripening as yield becomes proportionally more limited than vegetative growth. Under such circumstances, a N deficiency carries two quite negative features: from one side it severely limits yield with obvious negative impacts on business sustainability and, from the other side, it will foster a mechanism—i.e. too accelerated ripening—that is already one of the main concerns related to global warming.

### *Author contributions Statement*

CS: Data curation, formal analysis, writing – original draft; BM-M: Data curation, formal analysis, Writing - original draft; MG: Data curation and review; AG: Data curation and formal analysis; SP: Supervision, writing - review & editing; LL: Data curation, writing - review & editing; MT: Supervision, review & editing.

## Supplementary Information

Below is the link to the electronic supplementary material.

Supplementary file1 (XLSX 1680 KB)
